# The effects of aging on biosynthetic processes in the rat hypothalamic osmoregulatory neuroendocrine system

**DOI:** 10.1016/j.neurobiolaging.2018.01.008

**Published:** 2018-05

**Authors:** Michael P. Greenwood, Mingkwan Greenwood, Elena V. Romanova, Andre S. Mecawi, Alex Paterson, Olivera Sarenac, Nina Japundžić-Žigon, José Antunes-Rodrigues, Julian F.R. Paton, Jonathan V. Sweedler, David Murphy

**Affiliations:** a School of Clinical Sciences, University of Bristol, Bristol, England; b Department of Chemistry and the Beckman Institute for Advanced Science and Technology, University of Illinois at Urbana-Champaign, Urbana, IL, USA; c School of Medicine of Ribeirão Preto, University of São Paulo, Ribeirão Preto, Brazil; d Department of Physiology, University of Malaya, Kuala Lumpur, Malaysia; e Department of Physiological Sciences, Institute of Biological and Health Sciênces, Federal Rural University of Rio de Janeiro, Seropedica, Brazil; f Institute of Pharmacology, Clinical Pharmacology and Toxicology, Faculty of Medicine, University of Belgrade, Belgrade, Serbia; g School of Physiology and Pharmacology, University of Bristol, Bristol, England

**Keywords:** Aging, Vasopressin, Supraoptic nucleus, Methylation, Gene expression, Peptidomics

## Abstract

Elderly people exhibit a diminished capacity to cope with osmotic challenges such as dehydration. We have undertaken a detailed molecular analysis of arginine vasopressin (AVP) biosynthetic processes in the supraoptic nucleus (SON) of the hypothalamus and secretory activity in the posterior pituitary of adult (3 months) and aged (18 months) rats, to provide a comprehensive analysis of age-associated changes to the AVP system. By matrix-assisted laser desorption/ionization time-of-flight mass spectrometry analysis, we identified differences in pituitary peptides, including AVP, in adult and aged rats under both basal and dehydrated states. In the SON, increased Avp gene transcription, coincided with reduced Avp promoter methylation in aged rats. Based on transcriptome data, we have previously characterized a number of novel dehydration-induced regulatory factors involved in the response of the SON to osmotic cues. We found that some of these increase in expression with age, while dehydration-induced expression of these genes in the SON was attenuated in aged rats. In summary, we show that aging alters the rat AVP system at the genome, transcriptome, and peptidome levels. These alterations however did not affect circulating levels of AVP in basal or dehydrated states.

## 1 Introduction

As we age, disorders of body salt and water composition become more commonplace. Cases of hyponatremia/hypernatremia are much more prevalent in the elderly, where they have been linked to increased incidences of falls, fractures, and osteoporosis, thus contributing to increased hospital admissions and morbidity and mortality (Cowen et al., 2013). To promote healthy living well into old age, it is thus necessary to determine why such imbalances occur. Age-associated changes to both peripheral and central mechanisms that control salt and water homeostasis are deemed responsible. There is a progressive age-related decline in renal function, with less urine concentrating capacities in the elderly compared with younger subjects (Ishunina and Swaab, 2002). Such impaired capacity to conserve body water, together with reports of reduced thirst and inadequate fluid intake after periods of fluid deprivation, makes the elderly more susceptible to dehydration (Mack et al., 1994, Phillips et al., 1993). Inappropriate release of the antidiuretic hormone arginine vasopressin (AVP) into the systemic circulation has been highlighted as one of the causes of irregular water homeostasis in aging (Swaab and Bao, 2011).

AVP is synthesized in magnocellular neurons of the supraoptic nucleus (SON) and paraventricular nucleus (PVN) of the hypothalamus. A change in plasma osmolality is detected by osmosensitive neurons in circumventricular organs of the brain that provide direct inputs to shape the firing of AVP magnocellular neurons that are osmosensitive themselves and to coordinate AVP synthesis and secretion from the posterior (neural) lobe of the pituitary gland (Mecawi Ade et al., 2015, Nissen et al., 1994, Zhang and Bourque, 2003). Once released, following incidences such as rise in plasma osmolality or decrease in blood volume (Kondo et al., 2004), AVP promotes sodium and water reabsorption by the kidney (Ares et al., 2011, Breyer and Ando, 1994). When placed under stress, the capabilities of the AVP system have been shown to decrease with age (Frolkis et al., 1999, Keck et al., 2000, Sladek et al., 1981).

The AVP system has been interrogated on multiple levels, from synthesis to secretion, in aged subjects with differing results. For example, basal circulating AVP levels have been found to decrease, to remain unchanged, and to increase with age in humans, as well as rodents (Frolkis et al., 1999). These discrepancies have been attributed to genetic, age, and strain differences. Although there are many disputations, 1 area of agreement is that, while in many brain areas neuronal activity decreases with age (Burke and Barnes, 2006), paradoxically AVP neurons become more active (Palin et al., 2009, Terwel et al., 1992). This hyperactivity is thought to be a compensatory mechanism for decreased responsiveness to AVP in the kidney due to decreased receptor abundance in aged subjects (reviewed by Ishunina and Swaab, 2002), although this theory has been questioned (Preisser et al., 2004) and is not intuitive with the profound difference in circulating AVP described in aging models. The AVP magnocellular neurons undergo numerous morphological changes as they age including increased size of perikarya, nucleoli, and Golgi apparatus in humans and rodents (Ishunina and Swaab, 2002), analogous to morphological changes in these neurons with dehydration (Hatton and Walters, 1973). Increased AVP neuron size in states of dehydration is recognized as a necessary measure to meet cellular demands for increased transcription and protein synthesis under hypertonic stimulation where circulating levels of AVP are robustly increased (Zhang et al., 2001). It has been suggested that such hyperactivity of AVP neurons may in itself lead to electrolyte disorders in the elderly (Swaab and Bao, 2011), but the relationship between the activity of AVP neurons and circulating levels of AVP is poorly understood.

Transcriptional changes have been identified in AVP neurons with aging. A study by Palin et al. (Palin et al., 2009) showed increased expression of immediate early gene c-Fos, a commonly used marker of neuronal activity, in the rat SON, consistent with hyperactivation of the AVP neurons. In contrast to increased activity under basal conditions, reports have described an attenuation of the evoked AVP secretion in response to osmotic stress with aging in rodents (Sladek and Olschowka, 1994, Swenson et al., 1997). This has led some to suggest that deficits in mechanisms controlling transcription, mRNA stability, or translation in the aging SON magnocellular neurons may be responsible (Lucassen et al., 1997). Moreover, we recently showed that dehydration initiates the formation of new methylation marks on the rat Avp promoter (Greenwood et al., 2016a), suggesting that altered methylation patterns could lie beneath these transcriptional changes in aging AVP neurons.

Few studies have sought to combine information on the physiological aspects of aging with analyses of the molecular changes occurring in the hypothalamus. The reasons why old AVP neurons have elevated basal activity, or why they can fail to adequately respond under stress, is not well understood. In particular, the molecular basis for these changes in relation to circulating levels of AVP has received little attention. We reasoned that aging-associated deficits in the AVP system may be due to a combination of changes at the genome, transcriptome, and peptidome levels, and that these changes might be responsible for disturbances in osmotic stability. In this study, we have interrogated the physiological aspects of aging, performed metabolic measurements, and analyzed peptide levels in plasma and pituitary, correlating the results with molecular events occurring within the hypothalamus of aged rats. Furthermore, we have used our extensive knowledge of the transcriptome of the adult rat SON in euhydrated and dehydrated states to uncover novel changes surrounding altered Avp transcription in aging.

## 2 Materials and methods

### 2.1 Animals

All experiments were performed under a Home Office UK licence held under, and in strict accordance with, the provisions of the UK Animals (Scientific Procedures) Act (1986); they had also been approved by the University of Bristol Animal Welfare and Ethical Review Board. We choose to use male Wistar Han rats from the international genetic standardization program (IGS) in our aging study (Charles River, France). The carefully managed breeding program for these animals helps to manage genetic drift, so colonies bred in different locations around the world are not significantly divergent from each other giving a level continuity in aging studies performed in laboratories worldwide. The Charles River Han Wistar rats have been extensively studied at 2 years of age when these rats are reaching the end of their natural life spans. Incidences of neoplastic and non-neoplastic lesions were high in tissues including the kidney and pituitary gland at this age. Furthermore, rats surviving to this age varied from 30% to 80% across 20 control studies (www.criver.com). Therefore, in this aging study, we opted for rats of 18 months of age to minimize pathophysiological effects and thus allow investigation of the aging process in healthy animals. All adult rats used in this study were free of pituitary tumors; however, 6 of 50 aged animals were removed from this study because of tumors on the pituitary gland. On arrival, rats were 2 weeks younger than the desired ages, 3 months (adult) and 18 months (aged), to enable sufficient time for acclimatization before experimentation. Rats were housed at a constant temperature of 22 °C and a relative humidity of 50%–60% (v/v) under a 14:10-hour light/dark cycle (lights on at 0500) with food and water *ad libitum* for 2 weeks. To induce hyperosmotic stress, both adult and aged rats were randomly assigned to 2 groups: control (free access to drinking water) and dehydrated (removal of drinking water for 3 days). All rats were humanely killed by striking of the cranium (stunning) and then immediately decapitated with a small-animal guillotine (Harvard Apparatus, Holliston, MA). Trunk blood was collected in heparin-coated tubes. Brains were rapidly removed from the cranium and immediately frozen by covering with powdered dry ice (within 3 minutes of stunning). The pituitary gland was removed from the base of the skull within 2 minutes after decapitation. The neurointermediate lobe (NIL) was carefully separated from the anterior pituitary using a scalpel blade and then either placed into 1.5-mL tubes containing 500 μL of 0.1 M of HCl or 0.2-mL tubes containing 150 μL of 15 mg/mL of 2, 5-dihydroxybenzoic acid (DHBA) solution. Frozen brains and NIL in HCl solution were stored at −80 °C, whereas NILs in DHBA solution were stored at 4 °C. Animal experiments were performed between 9 am and 2 pm.

### 2.2 Metabolic measures in adult and aged rats

For metabolic measurements, animals were individually housed in metabolic cages (Techniplast, Italy) to allow precise daily measures of fluid and food intake and urine output. A plastic gnawing disc was suspended from the lid of the cage to provide environmental enrichment throughout the study. Animals were first acclimatized to metabolic cages for 48 hours. Measures of food hoppers, water bottles, and urine collection tubes were performed for 3 consecutive days, by weight. Extrarenal secretion of fluid was calculated by subtracting water intake from urine output. Plasma and urine osmolalities were measured by freezing point depression using a Roebling micro-osmometer (Camlab).

### 2.3 Vasopressin measures

The NIL of the pituitary was sonicated for 15 seconds in 0.1 M of HCl and incubated at 85 °C for 20 minutes. Cellular debris was removed by centrifugation at 3000 × *g* for 30 minutes at 4 °C. Pituitary AVP content was determined using an AVP^8^-Vasopressin ELISA (ADI-900-017A; Enzo) kit. The supernatant was diluted (1:10,000) with assay buffer, and ELISA was performed following the manufacturer's protocol. The signal was detected on an iMark microplate absorbance reader (Bio-Rad). For radioimmunoassay, trunk blood was centrifuged at 1600 × *g* for 15 minutes at 4 °C. Extractions were performed from 1 mL of plasma. Two sample volumes of ice-cold acetone were added, and samples were vortexed for 1 minute. Protein precipitates were removed by centrifugation at 2500 × *g*, 4 °C, for 25 minutes. The supernatant was transferred to a new tube and mixed with 2 mL of cold petroleum ether by vortexing for 1 minute. The tubes were left to stand for 1 minute at room temperature before discarding the upper phase. The lower phase solution was lyophilized using a freeze dryer (Benchtop Pro; Biopharma). AVP concentration was determined by a specific radioimmunoassay (Husain et al., 1973).

### 2.4 Peptide analysis of the NIL

Peptides were measured directly in individual NIL extracts by mass spectrometry (MS) (Romanova et al., 2014, Romanova and Sweedler, 2015).

#### 2.4.1 Extraction of peptides

NIL samples were incubated in 15 mg/mL of DHBA solution for 48 hours as described by Romanova et al. (2008). The samples were grouped as follows: adult control (n = 14), adult 3-day dehydrated (n = 16), aged control (n = 11), and aged 3-day dehydrated (n = 13).

#### 2.4.2 Measurement of the NIL peptide profiles by MALDI-TOF MS

For MALDI-TOF MS measurements, 0.7 μL of the NIL extraction solution was spotted on a stainless steel MALDI target in triplicates and cocrystallized with 0.7 μL of a freshly prepared concentrated DHBA matrix (50 mg/mL, 50% (v/v) acetone). Positive ion mass spectra of each spotted sample were acquired automatically at 1-KHz laser frequency, and constant power optimized for the sample type in the 600–6000 *m/z* region using an ultrafleXtreme mass spectrometer (Bruker Daltonics) operated in reflectron mode via the AutoXecute protocol. Acquisition parameters included laser fuzzy control logic, random laser walk over the entire sample area, 250 laser shots per raster step, maximum of 5000 shots per sample in 250-shot increments, and dynamic termination of spectrum acquisition when the signal intensity reached 30,000 counts for 3 peaks, regardless of the number of fired laser shots. Peak evaluation was set to a signal intensity per shot of 20 or above, minimal resolution of 10,000, signal-to-noise ratio (S/N) = 3, maximum 300 peaks per spectrum, and centroid peak detection algorithm; 50 failed spectrum judgments were required before acquisition moving to the next sample. External quadratic calibration was adjusted automatically for every 5 × 5 sample spot square.

#### 2.4.3 Principal component analysis of the peptide profiles

Statistical analysis of raw MALDI MS data was performed using ClinProTools 2.2 software (Bruker Daltonics). All spectra were normalized to total ion count upon loading into ClinProTools and level scaled. Spectra were processed for convex hull baseline correction within 800–5500 *m/z*, smoothed with 0.1 Da × 2 cycles of the Savitzky-Golay method and a data reduction factor of 2, null spectra exclusion was enabled, and spectra grouping applied. Other criteria included automatic peaks selection on the total average group spectrum by intensity, S/N = 5 cut off, 1% relative threshold base peak on average group spectrum, unlimited picking. Manual peak editing for the integration area after automatic peak picking was done on the mean spectrum representative of each sample group to include entire isotopic clusters of highly resolved peaks. Peptide profiles of the mean spectra were compared by principal component analysis (PCA) followed by the Anderson-Darling normality test and *Student's unpaired t*-test for normal distributed data. Data not showing normal distributions (*p*_Anderson-Darling_ ≤ 0.05) were evaluated by Kruskal-Wallis tests, respectively (Kruskal and Wallis, 1952, Stephens, 1974, Wilcoxon, 1945). To decrease the number of false positives while computing individual peak statistics on the complex spectra, the Benjamini-Hochberg procedure incorporated into ClinProTools was automatically applied for *p*-value adjustment during analysis (Dudoit and Shaffer, 2003). Unsupervised clustering of spectra was performed on PCA-modified data using Euclidean distance, average distance methods, and a Minkowski exponent of 1.5. The following peptide profile differences were investigated: (1) between control aged and adult rats; (2) between dehydrated aged and adult rats; and (3) between control and dehydrated rats of either age.

### 2.5 Dual DNA and RNA extraction from SON punch samples

SON samples (12 unilateral punches) were collected from 12 coronal slices using a 0.35-mm sample corer (Fine Scientific Tools) using the optic chiasm as a reference. Total RNA and genomic DNA were extracted from each sample as previously described (Greenwood et al., 2016a).

### 2.6 Complementary DNA synthesis and quantitative PCR

For cDNA synthesis, 40 ng of total RNA was reverse transcribed using the QuantiTect reverse transcription (RT) kit (Qiagen). Primers for rat genes used in this study: Avp (5′-TGCCTGCTACTTCCAGAACTGC-3′ and 5′-AGGGGAGACACTGTCTCAGCTC-3′), heteronuclear Avp (hnAvp) (5′-GAGGCAAGAGGGCCACATC-3′ and 5′-CTCTCCTAGCCCATGACCCTT-3′), ras-related dexamethasone induced 1 (Rasd1) (5′-CCCTCAGCGTTGTGCCTACT-3′ and 5′-AAAGAGCGCACGGAACATCT-3′), caprin family member 2 (Caprin2) (5′-CAGGGTTAAGTGCAAGCGAT-3′ and 5′-CTGGTGGTTGACTGGTTGAG-3′), c-Fos (5′-AGCATGGGCTCCCCTGTCA-3′ and 5′-GAGACCAGAGTGGGCTGCA-3′), cAMP responsive element binding protein 3 like 1 (Creb3l1) (5′-GCCAACAGGACCCTGCTCCA-3′ and 5′-AGTGCCAGTCTGTGTGGCCG-3′), gonadotropin inducible ovarian transcription factor 1 (Giot1) (5′-GACACTTCCGGTCCGTCATAG-3′ and 5′-GCCTCACTCAAGCACCCAGT-3′), DNA methyltransferase 1 (Dnmt1) (5′-AACCACTCAGCATTCCCGTA-3′ and 5′-TGCTGGTACTTCAGGTCAGG-3′), Dnmt3a (5′-AAGACCCCTGGAACTGCTAC-3′ and 5′-TGGCGAAGAACATCTGGAGT-3′), mature oxytocin (Ot) (5′-TGCCCCAGTCTTGCTTGCT-3′ and 5′-TCCAGGTCTAGCGCAGCCC-3′), heteronuclear Ot (hnOt) (5′-TGAGCAGGAGGGGGCCTAGC-3′ and 5′-TGCAAGAGAAATGGGTCAGTGGC-3′), proprotein convertase subtilisin/kexin type 1 inhibitor (proSAAS) (5′-GAGCTGCTGAGGTACTTGCT-3′ and 5′-ACCCAAATCCTGGTCCACAG-3′), heteronuclear proSAAS (hnproSAAS) (5′-GAAGTGACGACCGAGGTGTA-3′ and 5′-GCAGTATTGTAGGGCGTTCG-3′), tet methylcytosine dioxygenase 1 (Tet1) (5′-TGACCCACTCTTACCAGACC-3′ and 5′-GATGGGCCATTGCTTGATGT-3′), Tet2 (5′-TCGGAGGAGAAGAGTCAGGA-3′ and 5′-TAGGGCTTGCATTTTCCATC-3′), Tet3 (5′-ATGGCATGAAACCACCCAAC-3′ and 5′-ACTTGATCTTCCCCTCCAGC-3′), and ribosomal protein L19 (Rpl19) (5′-GCGTCTGCAGCCATGAGTA-3′ and 5′-TGGCATTGGCGATTTCGTTG-3′) were synthesized by Eurofins MWG Operon. QuantiTect Primer Assays for solute carrier family 12, member 1 (Slc12a1) were purchased from Qiagen. The optimization and validation of primers were performed using standard Applied Biosystems protocols. The cDNA from RT reaction was diluted 1:4 with H_2_O and used as a template for subsequent polymerase chain reactions (PCRs), which were carried out in duplicate using SYBR green (Roche) on an Applied Biosystems StepOnePlus Real-Time PCR system. For relative quantification of gene expression, the 2^−ΔΔCT^ method was used (Livak and Schmittgen, 2001). The internal control gene used for these analyses was the housekeeping gene Rpl19.

### 2.7 Poly(A) tail–length assay

The poly(A) tail length of the Avp mRNA was examined using the USB poly(A) Tail–Length Assay Kit (Affymetrix). RNA extracted from SON (50 ng) was used as the starting material. Guanosine and inosine residues were added to the 3′ ends of poly(A)-containing RNAs using the poly(A) polymerase enzyme. After incubation at 37 °C for 1 hour, stop solution was added and the tailed-RNAs were converted to cDNA by RT using the newly added G/I tails as priming sites. PCR amplification products were generated by using 2 primer sets: set 1, gene-specific forward and reverse primer set for Avp (forward 5′-CGAGTGTCGAGAGGGTTTTT-3′, reverse 5′-TTTATTTTCCATGCTGTAGG-3′), and set 2, Avp gene–specific forward primer and a universal reverse primer provided in the kit. PCR reactions were performed using 2 μL of the undiluted RT sample. PCRs were performed using the following cycling conditions: 94 °C for 2 minutes followed by 40 cycles of 94 °C for 10 seconds, 60 °C for 45 seconds, and 72 °C for 5 minutes. The PCR products were separated on 2.5% (w/v) agarose/TAE gel. The PCR products were visualized on ethidium bromide–stained gels using a Syngene G:BOX imaging system.

### 2.8 Bisulfite conversion and sequencing

Genomic DNA from SON punches (25 ng) was bisulfite converted using an EZ DNA Methylation-Gold kit (Zymo Research). The amplification and sequencing steps were performed as previously described (Greenwood et al., 2016a).

## 3 Results

### 3.1 Physiological assessment of adult and aged rats

**Fig. 1:**
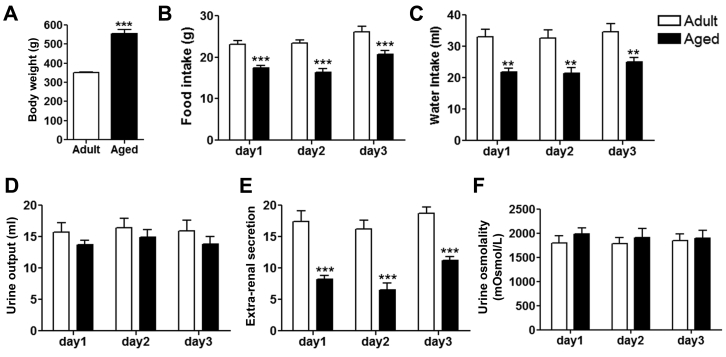
Comparison of metabolic parameters in adult and aged rats. Physiological parameters—(A) body weight, (B) food intake, (C) water intake, (D) urine output, (E) extrarenal water secretion, and (F) urine osmolality—were recorded in adult (3-month old) and aged (18-month old) male rats (n = 6) housed in metabolic cages. **, *p* < 0.01; ***, *p* < 0.001 by the 2-way analysis of variance with Bonferroni post hoc test.

We singly housed adult and aged rats in metabolic cages to assess their ingestive behaviors. As expected, the average weight of aged rats was significantly higher than that of adult rats (Fig. 1A). Despite their larger size, aged rats consumed significantly less food (Fig. 1B) and water (Fig. 1C) over consecutive 24-hour periods compared to adult rats. The lower water intake in aged rats was not accompanied by a significant decrease in urine output compared to adult rats (Fig. 1D) but reflected a decrease in extrarenal water loss compared to their younger counterparts (Fig. 1E). Urine osmolality was not affected by age (Fig. 1F).

### 3.2 The AVP system in adult and aged rats

**Fig. 2:**
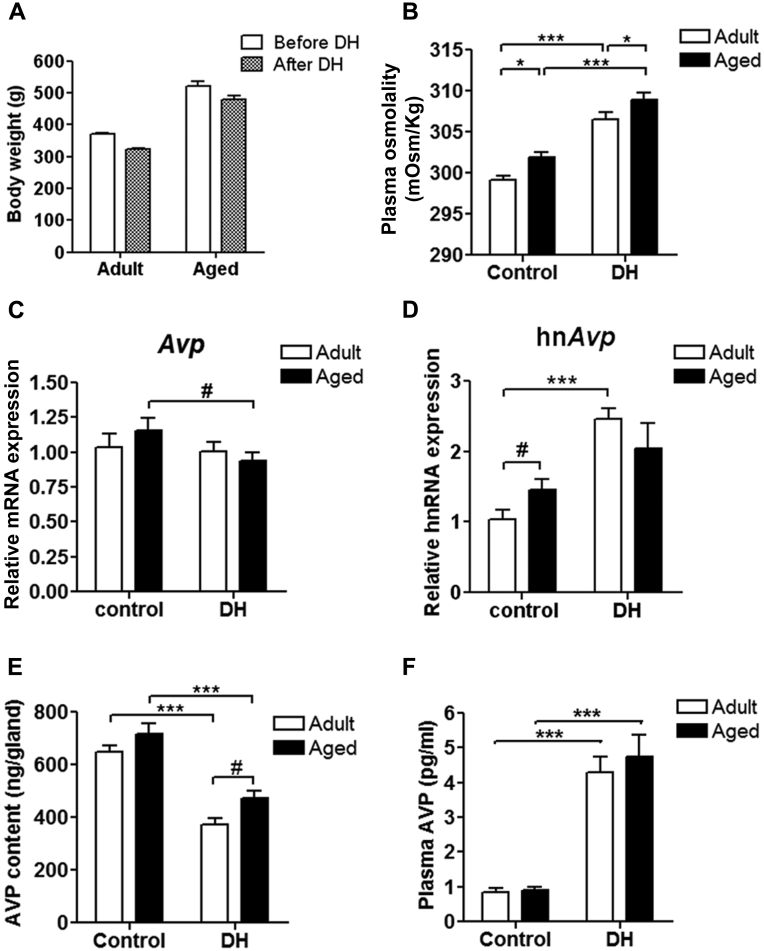
Effect of aging on the rat AVP system. Adult and aged male rats were subjected to dehydration for 3 days and AVP measures were performed. (A) Body weights were recorded before and after dehydration (n = 11–14). (B) Plasma osmolality was measured (n = 19–23) by freezing point depression. The effect of aging on Avp expression was examined at the transcriptional level in control and dehydrated adult and aged rats. (C, D) The RNA expression level of Avp (both heteronuclear [hnAvp] and mature form) was examined by qRT-PCR (n = 6). AVP measures were performed on NILs and plasma extracts. (E) AVP content in NILs was measured by ELISA (n = 9–10). (F) Plasma AVP level was determined by radioimmunoassay (n = 9). *, *p* < 0.05; ***, *p* < 0.001 by the 2-way analysis of variance with Bonferroni post hoc test. ^#^, *p* < 0.05 by unpaired *t*-test. Abbreviations: AVP, arginine vasopressin; DH, dehydrated; NIL, neurointermediate lobe.

To test how fluid homeostatic systems respond to osmotic stress, rats were deprived of water for 3 days. A decrease in weight from the starting body weight was observed for both adult and aged rats (Fig. 2A). This period of dehydration increased plasma osmolality by similar degrees in both aged and adult rats (Fig. 2B). However, a higher basal plasma osmolality was observed in aged rats, a difference that was preserved in response to 3 days of dehydration, suggestive of different osmolality set points in adult and aged rats. We investigated the expression of Avp mRNA and hnAvp RNA, a surrogate measure of Avp transcription (Herman et al., 1991), in the SON of control and dehydrated adult and aged rats using qRT-PCR (Fig. 2C and D). The abundance of Avp mRNA under basal conditions was not influenced by age (Fig. 2C), while increased hnAvp expression in aged animals indicated increased transcription of the Avp gene compared to adult rats (Fig. 2D). In contrast, the osmotic stimulus of dehydration increased hnAvp levels above adult basal measures for both adult and aged rats; however, this response was only significant in adult rats. The AVP content in the pituitary was investigated by AVP ELISA (Fig. 2E). There was a decrease in NIL AVP content in rats subjected to dehydration for both age groups. AVP NIL content was unchanged by age. Interestingly, the expected decline of AVP content with dehydration was marginally attenuated in aged rats, with higher AVP levels detected in aged dehydrated compared to adult dehydrated rats. However, there was no significant effect of age on basal or osmotically induced plasma AVP levels when comparing adult and aged rats (Fig. 2F).

### 3.3 Peptide analysis of the NIL in control and dehydrated states

#### 3.3.1 Effect of dehydration on NIL peptide profiles in adult rats

**Fig. 3:**
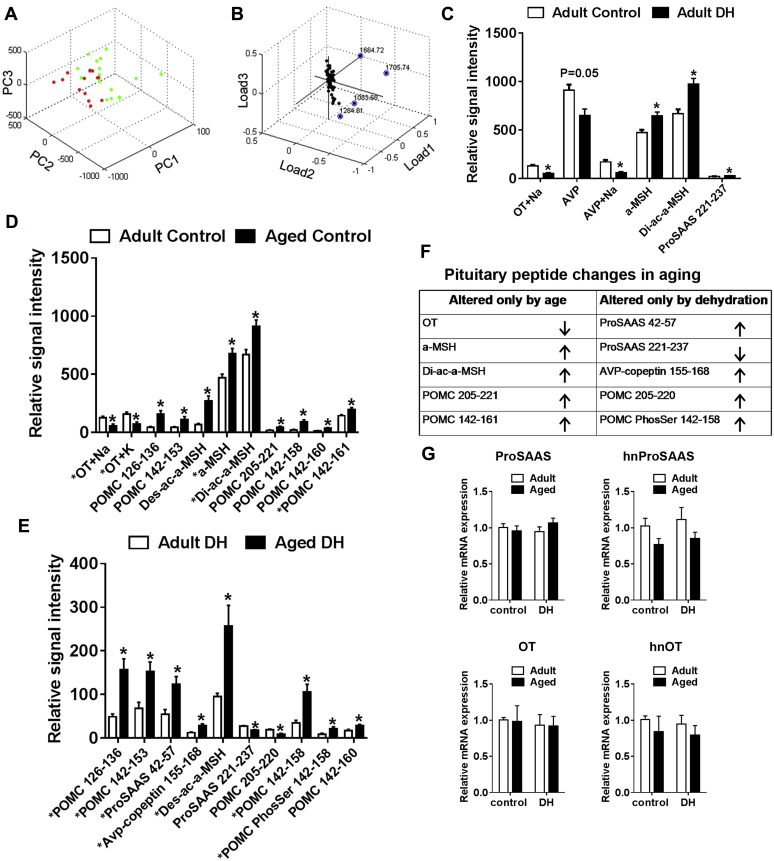
Peptide analysis in aged rats. Adult and aged male rats were subjected to dehydration for 3 days, and NIL peptide measures were performed by MALDI-TOF MS. (A–E) Signal intensity of peptides in NILs was measured by MALDI-TOF MS in individual tissue extracts from adult and aged rats in basal and dehydrated states (n = 11–16). (A) Control and dehydrated peptide profiles are easily classified by PCA according to PC1 (∼60% of variance) in adult rats; PCA plot is shown for the first 3 PCs. (B) Loading plot indicates that a small subset of peptides contributes to differences between control and dehydrated adult rats, among which are masses matching AVP, acetylated alpha-MSH, and di-acetylated alpha-MSH. (C) The AVP and OT signal decreases significantly, while the alpha-MSH signals and proSAAS (221-237) increase significantly with dehydration in adult rats. (D, E) The effect of age on peptide signals in the NIL. (D) The OT signals decreased, while the signals of POMC-derived peptides increased in aged compared to adult rats. (E) Peptide signals in the NIL of adult and aged rats subjected to 3 days of dehydration. The AVP-copeptin signal increased, whereas the proSAAS signal decreased with age. (F) Peptides uniquely altered as a function or aging or by just dehydration. (G) Relative RNA expression of proSAAS and OT in the SON of adult and aged rats. *p < 0.05 by unpaired *t*-test or peptide names beginning with * by Kruskal-Wallis tests. Abbreviations: AVP, arginine vasopressin; DH, dehydrated; NIL, neurointermediate lobe; PCA, principal component analysis; POMC, proopiomelanocortin.

The abundance of peptides in the NIL provides a good measure for assessing changes in peptide synthesis/secretion. To study effects of aging on peptide profiles in the NIL, we used MS-based peptide measurements (Romanova et al., 2013, Romanova and Sweedler, 2015) to characterize and quantify the neuropeptide changes of the NIL. In adult rats, control and dehydrated profiles were easily classified by PCA according to principal component 1 (PC1) (∼60% of variance; Fig. 3A). Loading plots indicated that peptides contributing to this difference matched the masses of AVP and its sodiated ion, the sodiated ion of oxytocin (OT), acetylated alpha-MSH, di-acetylated alpha-MSH, a portion of the ACTH domain, and other proopiomelanocortin (POMC)-derived peptides (Fig. 3B). The level of AVP and OT decreased with dehydration, whereas alpha-MSH and proSAAS levels increased (Fig. 3C).

#### 3.3.2 Comparison of the NIL peptide profiles between aged and adult rats

A comparison was performed for adult and aged rats. In PCA, 10 PCs were required to explain 93% of variance in the data set, with most sample segregation achieved along PC1 accounting for about 30% of variance. Spectra from aged animals showed more broad distribution within the 3D space constructed of the first 3 PCs. In this data set, a total of 78 peaks passed the criteria for statistics selection, of which 22 (∼30%) were detected at statistically different intensities (*p* ≤ 0.05) between compared age groups (see Supplementary Tables 1 and 2). Some of the peaks can be matched to POMC by peptide mass fingerprinting or other previously reported neuropeptides expressed in pituitary including OT (Fig. 3D). Relative to adult rats, the aged rats exhibited significant decrease in the sodiated ion and potassiumated ion of OT and increases in the intensity of peptides matching the masses of alpha-MSH, acetylated alpha-MSH, di-acetylated alpha-MSH, and 5 other POMC-derived peptides.

#### 3.3.3 Effect of aging on NIL peptide profiles in dehydrated rats

A comparison was performed for NILs of adult and aged dehydrated rats. In PCA, 13 PCs were required to explain 95% of variance in the data set. Both adult and aged rats showed a range of profiles that could not be reliably classified by PCA and unsupervised clustering. Four of 13 adult rats and 2 out of 16 aged rats were particularly different. A total of 121 peaks were selected for statistics, of which 20 (∼16%) were detected at statistically different intensities (*p* ≤ 0.05) between compared age groups (see Supplementary Tables 3 and 4). Similar to the control groups, aged animals had higher levels of peptides matching by mass to the POMC prohormone as well as proSAAS and AVP-copeptin (Fig. 3E).

#### 3.3.4 Effect of dehydration on NIL peptide profiles in aged rats

With the set of 24 aged rats (11 control and 13 dehydrated), no significant changes in NIL profiles were seen with dehydration, and no clear segregation was observed on a PCA plot.

#### 3.3.5 Peptide changes specific to age or dehydration

A number of peptides profiles in the NIL were altered only as a function of aging (Fig. 3F). In addition, a separate cohort of peptides was found to only differ between adult and aged rats in dehydration. (Fig. 3F). These included OT (aging) and proSAAS (dehydration) whose precursor proteins are known to be synthesized in magnocellular neurons in the SON (Murphy et al., 2012). Using qRT-PCR, we show that expression of these genes in the SON is not altered by age or dehydration (Fig. 3G).

### 3.4 Changes in Avp promoter methylation as a consequence of aging

**Fig. 4:**
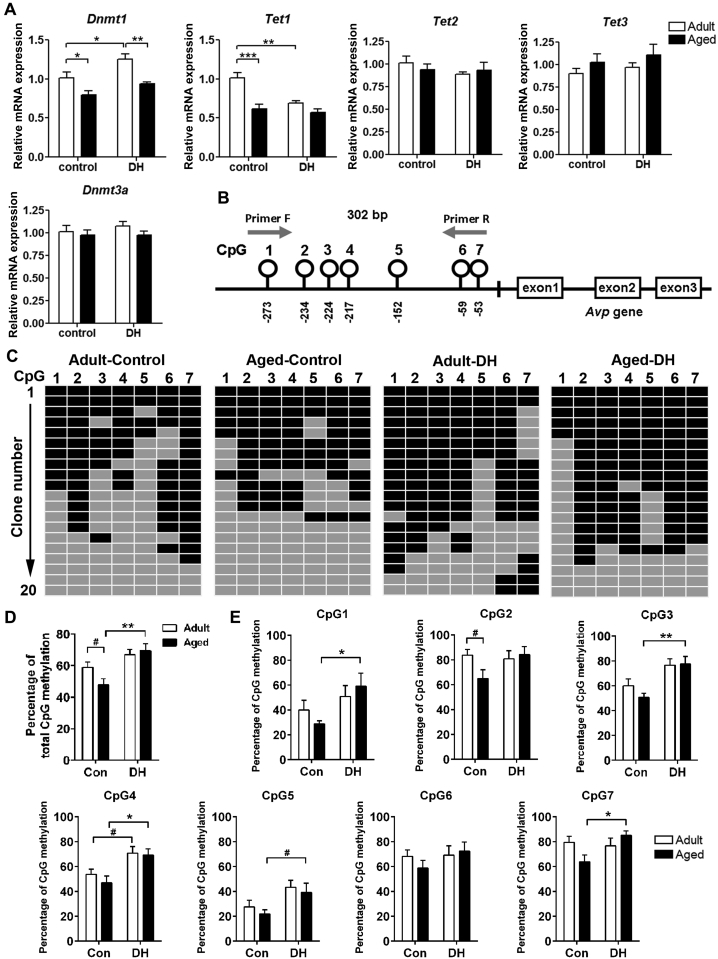
Epigenetic changes in Avp gene promoter in aging. Methylation status of the Avp promoter in the SON of control and dehydrated adult and aged male rats. (A) Relative mRNA expression of Dnmt1, Dnmt3a, Tet1, Tet2, and Tet3 was determined by qRT-PCR. (B) Diagram showing 7 CpG sites on the Avp promoter that were examined by colony-based PCR. (C) Representative tile diagrams showing the methylation status of 7 CpG sites for individual clones of the Avp promoter extracted from the SON. (D) Percentage of global methylation on this region of the Avp promoter in control and dehydrated adult and aged rats. (E) Percentage methylation of individual CpG sites on the Avp promoter in control and dehydrated adult and aged rats. *, *p* < 0.05; **, *p* < 0.01; ***, *p* < 0.001 by the 2-way analysis of variance with Tukey's post hoc test. ^#^, *p* < 0.05 by unpaired *t*-test. Abbreviation: SON, supraoptic nucleus.

To see if changes in methylation could account for Avp gene transcriptional differences in the SON with age, we looked at the expression of genes known to regulate methylation status of DNA, namely the Dnmt and Tet families, in the SON (Fig. 4A). We found decreased Dnmt1 and Tet1 in the SON of aged compared to adult rats, whereas expression of the closely related genes Dnmt3a and Tet2/3 remained unchanged with age. In the dehydrated state, Dnmt1 increased and Tet1 decreased in adult rat SON samples, whereas no changes in these genes were observed with dehydration and aging. To analyze gene-specific methylation changes, we chose to examine the methylation profile of the Avp promoter within the SON by sequence analysis of bisulfite-converted DNA. Using primers spanning the proximal Avp promoter (−325 to −24 bp), we investigated the methylation status of a cluster of 7 cytosine-phosphate-guanine (CpG) sites (Fig. 4B). Analyses of the methylation pattern of CpGs in single clones from individual control and dehydrated animals with aging are depicted in Fig. 4C. Analysis of the overall methylation of the Avp promoter for the SON revealed decreased methylation in aged compared to adult animals by the 2-way analysis of variance (*p* < 0.002), whereas methylation levels increased in response to dehydration in aged rats (Fig. 4D). In comparison, overall methylation was not significantly altered by dehydration in the SON of adults.

We next compared the methylation profiles of individual CpGs (Fig. 4E). Of the 7 CpGs analyzed, only CpG2 was significantly influenced by age, with lower methylation compared to adult controls. In aged rats, dehydration increased methylation of CpGs 1, 3–5, and 7 compared to aged controls. By contrast, only CpG4 showed increased methylation in dehydrated adult rats compared to adult controls. Of note, the methylation of individual CpGs was found to be similar in dehydrated adult and aged rats.

### 3.5 Aging changes gene expression in the SON and alters the effect of dehydration

**Fig. 5:**
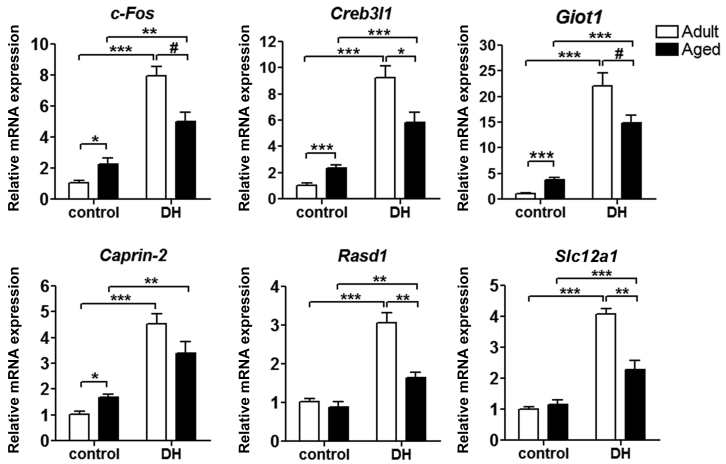
Effect of aging on the expression of osmotically induced genes. Relative mRNA expression of genes involved in hyperosmotic stress in the SON of the hypothalamus in control and 3-day dehydrated adult and aged rats. *, *p* < 0.05; **, *p* < 0.01; and ***, *p* < 0.001 by the 2-way analysis of variance with Bonferroni post hoc test. ^#^, *p* < 0.05 by unpaired *t*-test. Abbreviations: DH, dehydrated; SON, supraoptic nucleus.

We have used transcriptomics to catalog all of the genes expressed in the adult male SON and to identify genes that are differentially regulated by dehydration (Hindmarch et al., 2006). The challenge now is to place these genes into physiologically relevant pathways; thus, in pursuit of this aim, our functional investigations have revealed novel genes involved in AVP elaboration [Creb3l1 (Greenwood et al., 2014, Greenwood et al., 2015a, Greenwood et al., 2015b); Slc12a1 (Konopacka et al., 2015b); Caprin2 (Konopacka et al., 2015a); Giot1 (Qiu et al., 2007); and Rasd1 (Greenwood et al., 2016b)]. We have now asked if the expression of these genes is altered with aging, under both euhydrated and dehydrated conditions (Fig. 5). We used qRT-PCR to reveal age-related increases in mRNA expression of transcription factors c-Fos (a general marker of neuronal activation), Creb3l1, Giot1, and RNA-binding protein Caprin2 under basal conditions, while levels of the small G-protein Rasd1 and the Na-K-2Cl cotransporter Slc12a1 were unchanged. The expression of all of these genes was increased by dehydration in both adult and aged animals. In aged rats, dehydration induced smaller rises in the expression of all analyzed genes, reaching statistical significance compared to adult dehydrated rats, with 1 notable exception, Caprin2.

### 3.6 Post-transcriptional modification to Avp mRNA in aging

**Fig. 6:**
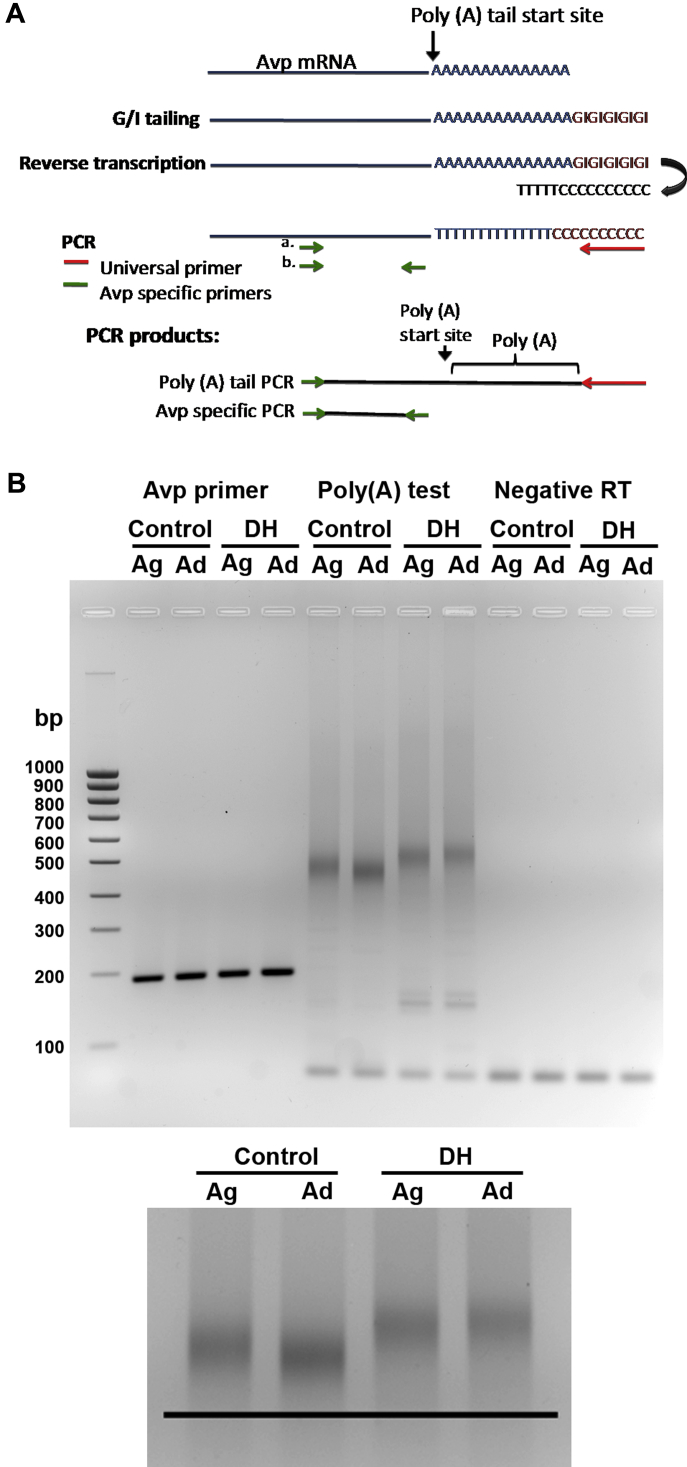
Effect of aging on transcriptional and post-transcriptional Avp gene expression. The effect of aging on Avp poly(A) tail length in the SON in control and dehydrated adult and aged rats. (A) Diagram of poly(A) tail assay design for the rat Avp gene. (B) poly(A) tail length of the Avp mRNA was examined using a PCR-based poly(A) tail assay. Abbreviations: Ad, adult; Ag, aged; DH, dehydrated; RT, reverse transcription; SON, supraoptic nucleus.

A known feature of the Avp mRNA is that the unusually long 3′ poly(A) tail further increases in length in response to osmotic stress (Carter and Murphy, 1991). Here, we have used poly(A) tail assays to determine the length of the Avp mRNA poly(A) tail in adult and aged rats in the basal condition and in response to dehydration (Fig. 6A). The length of the Avp poly(A) tail was found to be susceptible to change with aging. In aged rats, the poly(A) tail was longer than that in adult control rats (Fig. 6B), perhaps suggesting altered transcript stability with age. The Avp poly(A) tail length increased more in adult rats in dehydration, but overall poly(A) tail lengths ended up being the same size in both dehydrated groups reflecting the smaller starting point in adult rats.

## 4 Discussion

With increased life expectancy, maintaining health and well-being into old age is becoming a priority, making the push toward understanding our aging homeostatic systems ever more pertinent. A decline of appetite accompanied by a reduction in daily fluid intake, as we observed in the rat, is common behavioral characteristics observed in the elderly (Kmiec, 2006, Phillips et al., 1993), suggesting that our rat model is of particular value to study metabolic changes related to aging. Comparisons of basal and dehydrated urine osmolalities in both age groups suggested that urine concentrating capacity and thus renal function are not impaired in our model at this age. Furthermore, AVP circulating levels were comparable in adult and aged rats in the basal state and in response to dehydration, suggesting no changes in hypothalamo-neurohypophysial system (HNS) responsiveness to osmotic and volume stimuli. Hence, the circulating levels of AVP and the renal response to it are adequate to concentrate urine in these aging rats.

Chronic dehydration depletes AVP stores in the posterior pituitary to meet necessary circulatory demands for AVP to facilitate increased water uptake by the kidney (Antunes-Rodrigues et al., 2014). The large stores of AVP in the posterior pituitary were depleted by comparable amounts in both age groups by dehydration. However, when compared to adult rats, aged rats had higher levels of pituitary AVP as a consequence of dehydration. We propose that increased basal AVP pituitary is responsible for this difference as opposed to ineffective stimulation of AVP secretion. This concept is consistent with the comparable increases in circulating levels of AVP after dehydration. Therefore, the ability to store and secrete adequate quantities of AVP in response to 3 days of dehydration was not compromised in these Han Wistar rats at this age. Taken together, these data show that changes to AVP secretion cannot account for the observed metabolic changes in aged compared to adult rats. The altered fluid intake and plasma electrolytes may represent changes to other systems coordinating salt and water balance. For example, the renin-angiotensin-aldosterone and atrial natriuretic peptide systems are known to be altered in rats and humans as a function of aging (El-Sharkawy et al., 2014, Pollack et al., 1997, Silver et al., 1993). However, any involvement of these systems in this particular aging model remains to be investigated.

A higher set-point for basal plasma osmolality in our aged model, one of the reported characteristics of aging in humans and rodents (McLean et al., 1992, Terwel et al., 1992), provides one possible explanation for AVP neuron hyperactivation in the basal state. A small rise in plasma osmolality of approximately 1% is normally sufficient to activate Avp transcription in magnocellular neurons of the SON and PVN, and these transcriptional events are well known to occur together with increased AVP secretion from the posterior pituitary in adult rats (Arima et al., 1999). The higher plasma osmolality in aged rats, being approximately 1% above adult rats, was indeed associated with increased transcription but not with increased secretion of AVP. In contrast, 3 days of dehydration, a well-characterized model for activating Avp transcription in the SON (Greenwood et al., 2014), increased AVP secretion but not Avp transcription in the aged group. This is despite a rise in plasma osmolality of greater than 2% by this osmotic stimulus.

The synthesis and secretion of AVP are normally twinned to maintain neurohypophysial homeostasis because AVP stores in the pituitary become depleted and need to be replenished with newly synthesized AVP (Murphy and Carter, 1990). Any delay in replenishing pituitary AVP stores to prestimulus levels might leave the system at greater risk from further hyperosmotic insults. The elderly living at care homes have been shown to have lower daily intakes of fluid than those living at home. Furthermore, elderly people with cognitive impairments such as dementia often forget to drink. These behavioral characteristics, coupled with reduced thirst perception in elderly people, greatly increase their risk of dehydration (El-Sharkawy et al., 2014). In elderly patients admitted to hospitals, hypernatremia has been associated with an increased mortality rate (Snyder et al., 1987). In addition, clinical studies of care home patients who develop acute illness and require hospital treatment reported that approximately 30% became markedly hypernatremic in hospitals (Cowen et al., 2013). The uncoupling of plasma osmolality and Avp transcription did not alter AVP secretion here in healthy aging rats but may become important in pathophysiological conditions if the rate of Avp transcription ever fails to meet secretory demands.

We next investigated if the observed uncoupling of synthesis and secretion was unique to AVP in the aging HNS. We revealed a cohort of peptides, in particular, POMC-derived peptides, N-terminal truncated form of copeptin, proSAAS, and OT in addition to AVP, which were susceptible to changes with age and also dehydration. POMC has been shown to be expressed in the pituitary intermediate lobe, with its expression altered by osmotic stimulation and changes in blood pressure (Felder and Garland, 1989, Pardy et al., 1990). In rats supplied with a drinking diet of 2% NaCl, POMC mRNA expression was shown to decrease in the intermediate lobe of the pituitary (Pardy et al., 1990). In relation to blood pressure, spontaneously hypertensive (SHR) rats have lower POMC expression in the intermediate lobe compared to Wistar Kyoto (WKY) rats. Lowering blood pressure in SHR rats with antihypertensive agents normalizes POMC expression in the intermediate lobe to that in the WKY rat (Felder and Garland, 1989). We show here age-related increases in an array of POMC-derived peptides in the NIL under basal and dehydrated conditions. How, or if, these POMC peptides contribute to age-related changes to physiology is currently not known.

Copeptin and AVP are derived from the same common precursor molecule. Copeptin is the C-terminal part of pro-AVP that is cleaved during processing and released with AVP into the circulation. The functions of copeptin are not known, but due to its higher stability in plasma, it is commonly used as surrogate measure for circulating levels of AVP (Christ-Crain and Fenske, 2016). Here, we have identified an N-terminal truncated form of copeptin in the NIL and further show that the abundance of this peptide increases in the dehydrated rat as a function of aging. This peptide has previously been identified in a peptidomic study of the rat SON (Bora et al., 2008). Therefore, increased abundance of this peptide in the NIL might suggest increased processing of pro-AVP in aging magnocellular neurons in response to dehydration. In support of this concept, a study using microdialysis probes to measure release patterns of AVP in the PVN and SON in the aging male Wistar rat showed an age-associated increase in AVP release in the PVN, though not in the SON (Keck et al., 2000).

ProSAAS and OT are known to be expressed in magnocellular neurons of the hypothalamus and the posterior lobe of the pituitary gland (Bora et al., 2008, Gouraud et al., 2007). The propeptide precursor ProSAAS is processed into a number of smaller peptides in the brain and pituitary including big SAAS, little SAAS, PEN, big LEN, and little LEN (Mzhavia et al., 2001). Here, we identify age-associated alterations in truncated forms of little SAAS (ProSAAS 42-57) and PEN (ProSAAS 221-237) in the dehydrated NIL. Interestingly, dehydration for 3 days also increases ProSAAS expression in the SON while decreasing ProSAAS expression in the NIL, suggesting that the ProSAAS peptide might be secreted (Gouraud et al., 2007), although this remains to be determined. The previous peptidomic study performed on rat SON samples also identified this cleavage of ProSAAS (42-57) and multiple PEN peptides in magnocellular neurons. One possibility for the involvement of ProSAAS in the regulation AVP is through its interactions with proprotein convertase 1. Pro-AVP is processed by proprotein convertase 1, and ProSAAS inhibits the activity of this convertase (Murphy et al., 2012). Therefore, changes in ProSAAS expression in aging might alter the processing and thus the availability of AVP.

OT is probably best known for its role in female reproduction but also functions as a natriuretic hormone that reduces sodium appetite and increases sodium excretion at the kidney (Verbalis et al., 1991). Circulating levels of OT increase in response to stimulation by osmotic stress, and this depletes pituitary stores of this peptide consistent with this study (Silverman et al., 1990). Our data suggest that OT stores are depleted in the NIL as a consequence of aging, in agreement with earlier studies of the aging HNS (Silverman et al., 1990, Zbuzek et al., 1988). Because OT and ProSAAS are synthesized in magnocellular neurons of the hypothalamus, such as AVP, we have also investigated their transcript abundance in the SON to assess this phenomena of uncoupling observed for AVP. We report no age-related effects on OT or ProSAAS transcription in the SON, although our data indicate altered peptide levels in the pituitary.

We have made significant steps to understanding the mechanism regulating AVP in the adult rat and have applied our understanding to the Avp transcriptional changes in this aging model. Based on our recent study where we described altered methylation patterns of the Avp promoter with dehydration, we hypothesized that altered methylation marks in the Avp promoter could be responsible. Cellular aging is closely associated with a decrease in expression of Dnmt1, an enzyme that stabilizes methylation marks on DNA (Casillas et al., 2003), as we observed here in the SON. This is thought to be 1 reason for hypomethylation of DNA sequences in rodents as well as humans and is consistent with hypomethylation of the Avp promoter in the aged rat SON. We also found lower levels of Tet1 expression in the aged rat and adult SON following dehydration. Tet1, by hydroxylation of 5hmc, has been shown to promote active demethylation of DNA in the rodent brain (Guo et al., 2011).

The decrease in Avp promoter methylation in aged rats may perhaps explain the increased Avp transcription in the aging SON. Many studies have shown that lower levels of promoter methylation correlate with increased gene transcription. We previously reported increased Avp transcription in hypothalamic 4B cells following demethylation by 5-aza-2′-deoxycytidine treatment, consistent with this hypothesis (Greenwood et al., 2016a). Individual CpG sites were largely unaffected by age, apart from CpG2, which resides close to a cAMP responsive element, which underwent hypomethylation with age. Methylation at cAMP response element sites has been shown to inhibit cAMP response element binding protein (CREB)– mediated transcription (Elliott et al., 2010, Zhang et al., 2005), so hypomethylation of this site may serve to enhance Avp promoter activation by CREB (Iwasaki et al., 1997). Interestingly, methylation signatures on this segment of the Avp promoter remained largely unchanged by dehydration in adult rats, differing from our findings in the Sprague Dawley rat (Greenwood et al., 2016a). We suggest this is due to strain differences. Nonetheless, dehydration induced the hypermethylation of CpG3, 4, and 7 in aged rats, as observed in adult Sprague Dawley rats, thus adding strength to the argument for a relationship between the methylation status of specific Avp promoter CpGs and Avp gene transcription.

To further aid our understanding of AVP neuron activity in aging, we looked at the expression of genes known to be robustly induced by osmotic stimuli, and whose functions have been the subject of interrogation by us in relation to AVP biosynthesis in the SON, and the overall regulation of fluid balance in the rat (Greenwood et al., 2014, Greenwood et al., 2016b, Konopacka et al., 2015a, Konopacka et al., 2015b, Qiu et al., 2007). We recently identified Creb3l1 as a putative transcription factor regulating the expression of the Avp gene (Greenwood et al., 2014). Therefore, in the basal condition, increased Avp transcription can perhaps be explained by the upregulation of Creb3l1 expression, and conversely, the attenuated Creb3l1 induction in aged dehydrated rats following osmotic stimulation may explain the reduced capacity to elevate Avp. The altered expression of genes regulating transcriptional events (c-Fos, Creb3l1, and Giot1) in basal and dehydrated states implies dramatic changes in the SON transcriptome with aging. These genes are all activated by cAMP pathways (Greenwood et al., 2015a, Qiu et al., 2007), suggesting that altered cAMP signaling may determine altered transcriptional responses in the aged SON. The source of these altered signaling responses is not known but may be a consequence of either altered inputs from the circumventricular organs due to changes in plasma osmolality (McKinley et al., 2004) or changes within the magnocellular neurons themselves.

It is interesting to note that basal Rasd1 and Slc12a1 expression levels were not influenced by age implying activation of these genes by separate signaling pathways not affected by age. Rasd1 is a member of the Ras family of small G-proteins, which is expressed in AVP magnocellular neurons of the PVN and SON, where increased circulating glucocorticoids and/or raised plasma osmolality induce its expression (Greenwood et al., 2016b). Interestingly, plasma corticosterone levels increase in aging rodents as a result of hyperactivity of the hypothalamo-pituitary-adrenal axis (Goncharova, 2013). At the same time, there is an age-associated decrease in the sensitivity of the hypothalamus, along with other brain nuclei, to glucocorticoids (Goncharova, 2013), which may account for the blunted increase in Rasd1 expression in dehydrated aged animals, despite changes to corticosterone levels. By lentiviral vector–mediated overexpression of Rasd1 in the SON, we recently showed that Rasd1 inhibits osmotically induced Avp transcription in this nucleus (Greenwood et al., 2016b). The aged dehydrated rats appear to have lost this dehydration-induced inhibitory input on Avp transcription in the aging SON.

The expression of Slc12a1 in magnocellular neurons of the SON and PVN is also known to be induced by chronic and acute osmotic stimulation (Konopacka et al., 2015b). We recently showed that lentiviral-mediated knockdown of Slc12a1 in these hypothalamic nuclei altered fluid homeostasis by increasing fluid intake and urine output during salt loading. Furthermore, the loop diuretics bumetanide and furosemide were found to inhibit gamma-aminobutyric acid-mediated excitation of AVP neurons and AVP release, respectively. Therefore, an altered abundance of Slc12a1 might be expected to alter neuronal activity in aging AVP neurons.

The Avp mRNA is subject to post-transcriptional modification in the form of an increase in the length of the poly(A) tail, as seen in the SON in response to osmotic challenges (Carter and Murphy, 1991) and as we show here by age. An increased poly(A) tail length is thought to reduce the degradation and increase the stability of many transcripts (Zeevi et al., 1982) and may be involved in the control of translation (Palatnik et al., 1984). We recently showed that the RNA-binding protein Caprin2 binds to the Avp mRNA and, in doing so, mediates an increase in the length of the poly(A) tail (Konopacka et al., 2015a). It is interesting to note that the expression of Caprin2 in the aging SON increases in parallel with the Avp mRNA poly(A) tail length. The increase in Avp mRNA poly(A) tail length in response to dehydration was not affected by age, as previously reported (Sladek and Olschowka, 1994), which was further corroborated by there being no difference in Caprin2 expression in dehydrated adult and aged rats. A previous Northern blot study on Fischer 344 rat SON samples reported no change in Avp poly(A) tail length with age (Sladek and Olschowka, 1994). We suggest that this discrepancy could be due either to strain differences or to the different methodological approaches used.

### 4.1 Conclusions

**Fig. 7:**
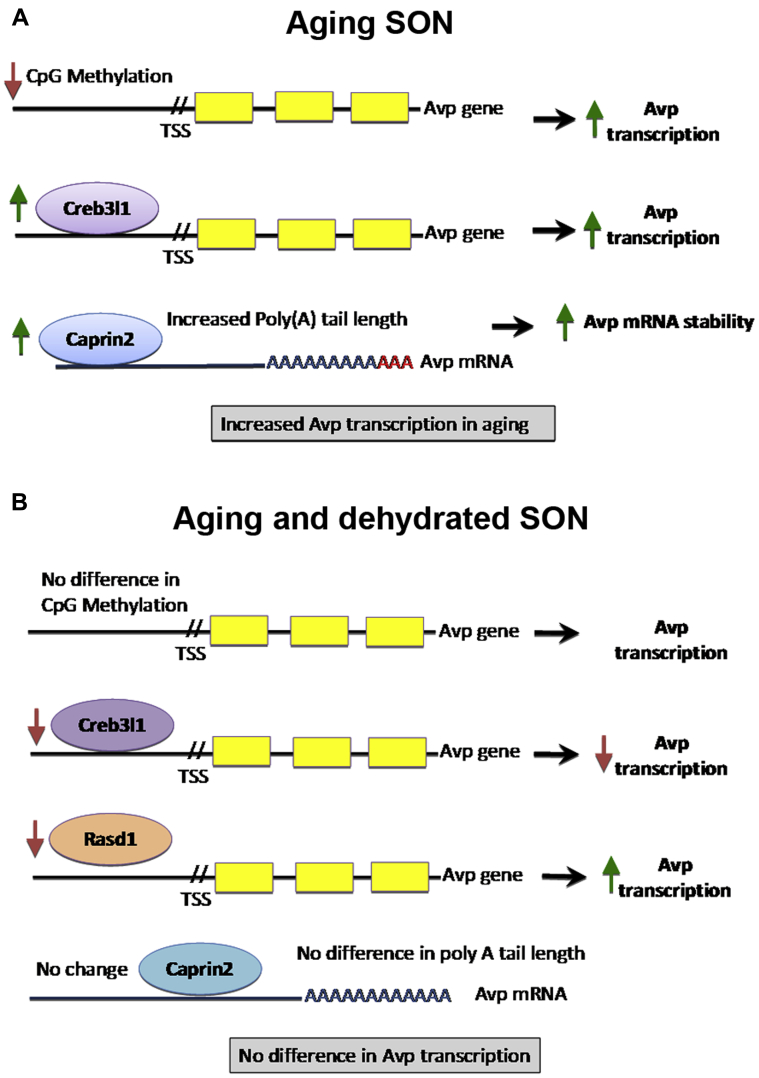
Modeling of molecular events in the SON in aging and dehydrated rats. (A) How aging may alter Avp expression in the SON compared to younger counterparts. (B) Proposed differential regulation of Avp expression in the SON as a function of both aging and dehydration. Abbreviation: SON, supraoptic nucleus.

In summary, we have performed a comprehensive analysis of the AVP system in aging Han Wistar rats. There were no age-related changes to AVP circulating levels in basal or dehydrated states, suggesting that the functioning of the HNS in body water homeostasis is intact in healthy rats at this age. In stark contrast, we describe in the magnocellular neurons of the SON a plethora of molecular changes known to alter Avp expression. These include methylation changes to the Avp promoter and altered expression of genes involved in transcriptional and post-transcriptional regulation of the Avp gene (Fig. 7). We currently do not understand the origin of these changes or why they are a necessary part of normal aging in the rat. The current rat model was perfectly capable of coping with 3 days of dehydration by secreting adequate quantities of AVP. However, this may not be true for pathophysiological conditions commonly encountered in the aging process. The stimulus secretion uncoupling of Avp transcription as seen here in normal aging could increase the likelihood of fluid and electrolyte disorders in critically ill elderly patients. Not being able to adequately correct or respond to fluid and electrolyte disturbances such as a rise plasma osmolality would certainly lead to hypernatremia, a condition that is regularly observed in elderly patients admitted to hospitals (El-Sharkawy et al., 2014). Further studies are necessary to address whether Avp transcription can be a rate-limiting step in the aging HNS. The identification by MS of additional peptides that are also influenced by age in the NIL gives further scope for exploring other peptidergic systems in the aging hypothalamus, in the context of healthy aging. Understanding how aging alters these hormonal systems may help to improve treatment regimens and perhaps improve the clinical outcomes for elderly patients.

## Disclosure statement

The authors have no actual or potential conflicts of interest.
