# Supplementary material for: The effects of aging on biosynthetic processes in the rat hypothalamic osmoregulatory neuroendocrine system
Source: Neurobiol Aging. 2018 May;65:178–91. doi: 10.1016/j.neurobiolaging.2018.01.008 (PMC5878011; doi:10.1016/j.neurobiolaging.2018.01.008)
Supplement: Supplementary Tables [file mmc1.pptx]

## Slide 1
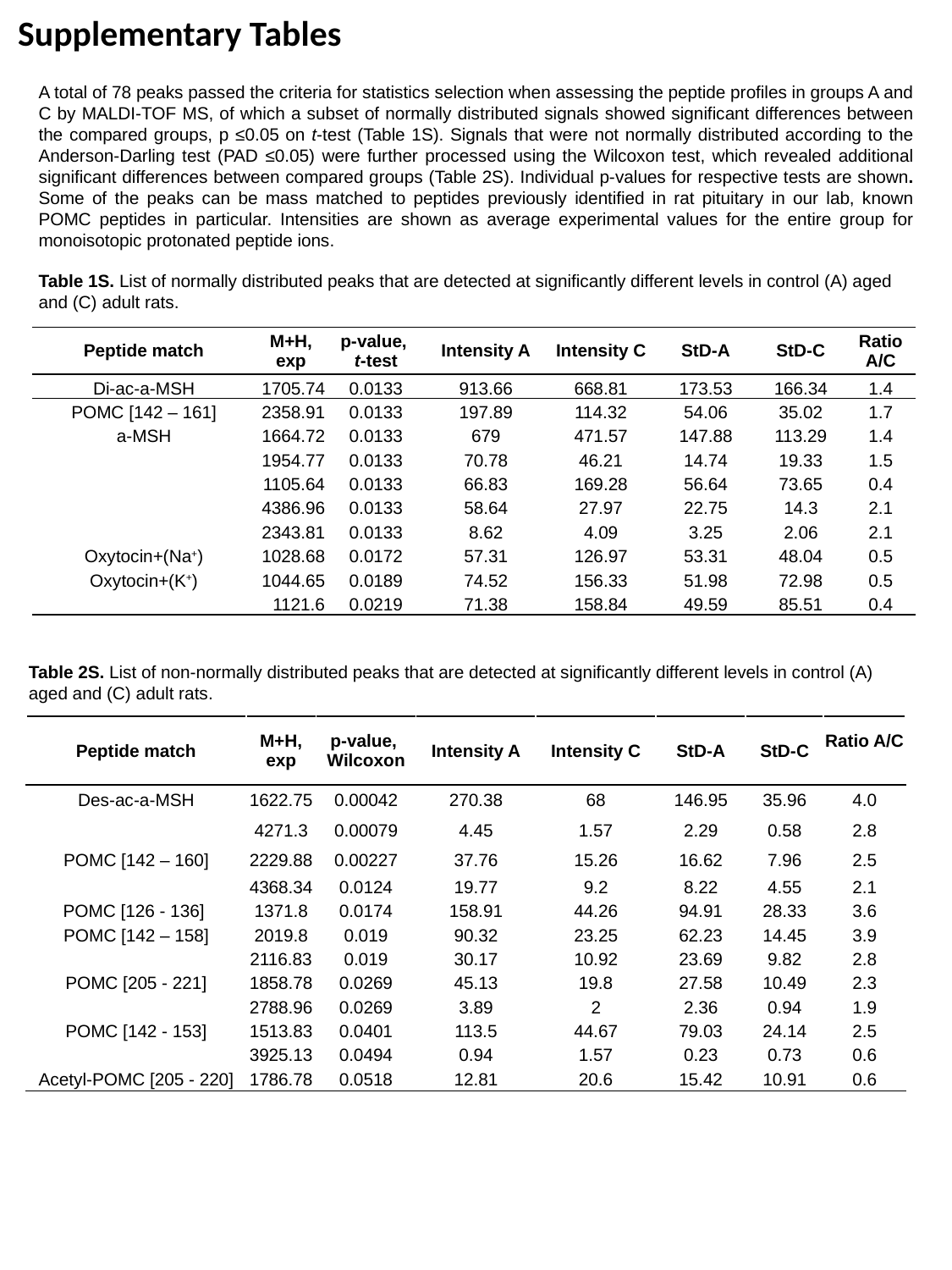

Supplementary Tables
A total of 78 peaks passed the criteria for statistics selection when assessing the peptide profiles in groups A and C by MALDI-TOF MS, of which a subset of normally distributed signals showed significant differences between the compared groups, p ≤0.05 on t-test (Table 1S). Signals that were not normally distributed according to the Anderson-Darling test (PAD ≤0.05) were further processed using the Wilcoxon test, which revealed additional significant differences between compared groups (Table 2S). Individual p-values for respective tests are shown. Some of the peaks can be mass matched to peptides previously identified in rat pituitary in our lab, known POMC peptides in particular. Intensities are shown as average experimental values for the entire group for monoisotopic protonated peptide ions.
Table 1S. List of normally distributed peaks that are detected at significantly different levels in control (A) aged and (C) adult rats.
| Peptide match | M+H, exp | p-value, t-test | Intensity A | Intensity C | StD-A | StD-C | Ratio A/C |
| --- | --- | --- | --- | --- | --- | --- | --- |
| Di-ac-a-MSH | 1705.74 | 0.0133 | 913.66 | 668.81 | 173.53 | 166.34 | 1.4 |
| POMC [142 – 161] | 2358.91 | 0.0133 | 197.89 | 114.32 | 54.06 | 35.02 | 1.7 |
| a-MSH | 1664.72 | 0.0133 | 679 | 471.57 | 147.88 | 113.29 | 1.4 |
| | 1954.77 | 0.0133 | 70.78 | 46.21 | 14.74 | 19.33 | 1.5 |
| | 1105.64 | 0.0133 | 66.83 | 169.28 | 56.64 | 73.65 | 0.4 |
| | 4386.96 | 0.0133 | 58.64 | 27.97 | 22.75 | 14.3 | 2.1 |
| | 2343.81 | 0.0133 | 8.62 | 4.09 | 3.25 | 2.06 | 2.1 |
| Oxytocin+(Na+) | 1028.68 | 0.0172 | 57.31 | 126.97 | 53.31 | 48.04 | 0.5 |
| Oxytocin+(K+) | 1044.65 | 0.0189 | 74.52 | 156.33 | 51.98 | 72.98 | 0.5 |
| | 1121.6 | 0.0219 | 71.38 | 158.84 | 49.59 | 85.51 | 0.4 |
Table 2S. List of non-normally distributed peaks that are detected at significantly different levels in control (A) aged and (C) adult rats.
| Peptide match | M+H, exp | p-value, Wilcoxon | Intensity A | Intensity C | StD-A | StD-C | Ratio A/C |
| --- | --- | --- | --- | --- | --- | --- | --- |
| Des-ac-a-MSH | 1622.75 | 0.00042 | 270.38 | 68 | 146.95 | 35.96 | 4.0 |
| | 4271.3 | 0.00079 | 4.45 | 1.57 | 2.29 | 0.58 | 2.8 |
| POMC [142 – 160] | 2229.88 | 0.00227 | 37.76 | 15.26 | 16.62 | 7.96 | 2.5 |
| | 4368.34 | 0.0124 | 19.77 | 9.2 | 8.22 | 4.55 | 2.1 |
| POMC [126 - 136] | 1371.8 | 0.0174 | 158.91 | 44.26 | 94.91 | 28.33 | 3.6 |
| POMC [142 – 158] | 2019.8 | 0.019 | 90.32 | 23.25 | 62.23 | 14.45 | 3.9 |
| | 2116.83 | 0.019 | 30.17 | 10.92 | 23.69 | 9.82 | 2.8 |
| POMC [205 - 221] | 1858.78 | 0.0269 | 45.13 | 19.8 | 27.58 | 10.49 | 2.3 |
| | 2788.96 | 0.0269 | 3.89 | 2 | 2.36 | 0.94 | 1.9 |
| POMC [142 - 153] | 1513.83 | 0.0401 | 113.5 | 44.67 | 79.03 | 24.14 | 2.5 |
| | 3925.13 | 0.0494 | 0.94 | 1.57 | 0.23 | 0.73 | 0.6 |
| Acetyl-POMC [205 - 220] | 1786.78 | 0.0518 | 12.81 | 20.6 | 15.42 | 10.91 | 0.6 |

## Slide 2
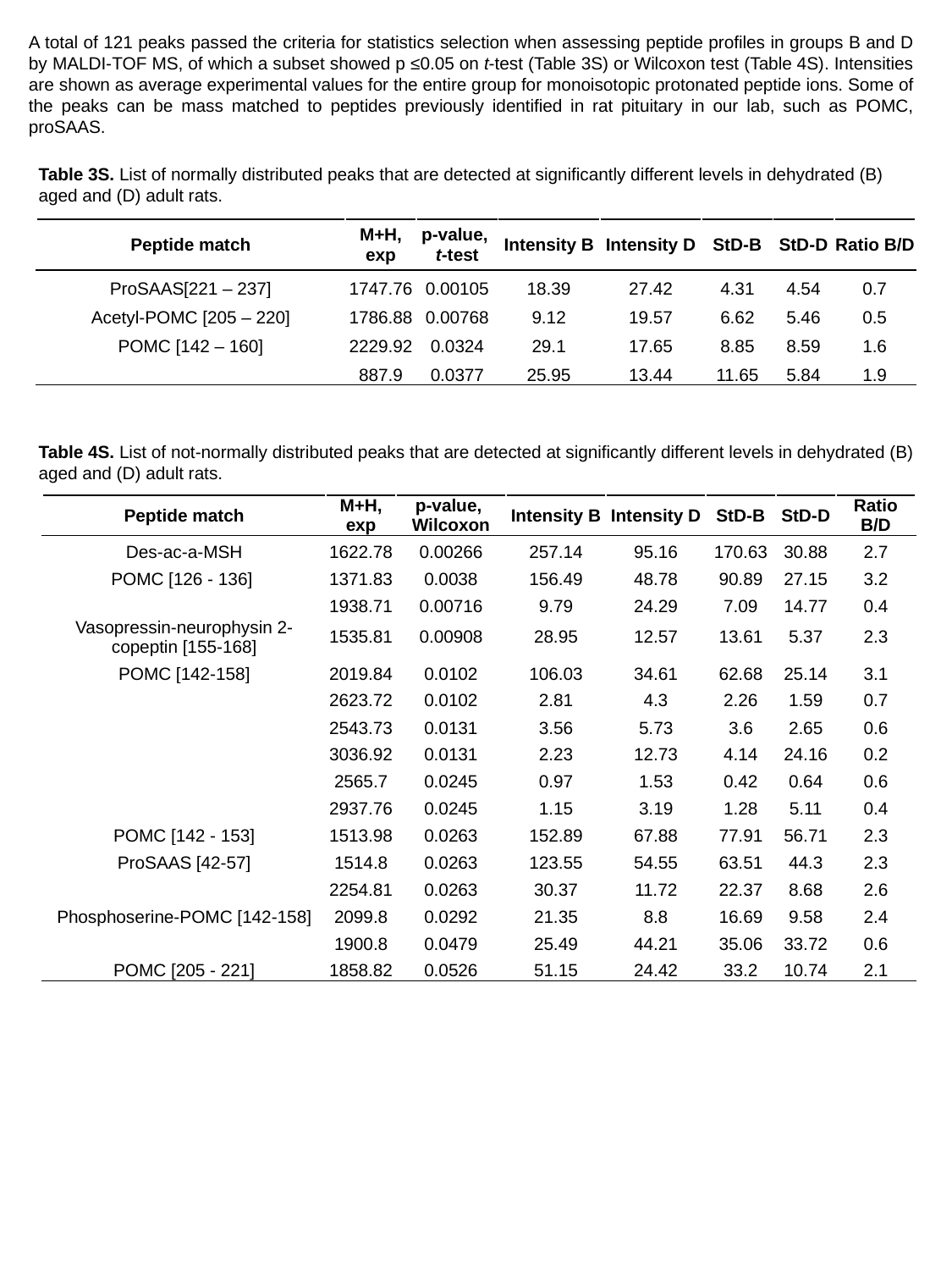

A total of 121 peaks passed the criteria for statistics selection when assessing peptide profiles in groups B and D by MALDI-TOF MS, of which a subset showed p ≤0.05 on t-test (Table 3S) or Wilcoxon test (Table 4S). Intensities are shown as average experimental values for the entire group for monoisotopic protonated peptide ions. Some of the peaks can be mass matched to peptides previously identified in rat pituitary in our lab, such as POMC, proSAAS.
Table 3S. List of normally distributed peaks that are detected at significantly different levels in dehydrated (B) aged and (D) adult rats.
| Peptide match | M+H, exp | p-value, t-test | Intensity B | Intensity D | StD-B | StD-D | Ratio B/D |
| --- | --- | --- | --- | --- | --- | --- | --- |
| ProSAAS[221 – 237] | 1747.76 | 0.00105 | 18.39 | 27.42 | 4.31 | 4.54 | 0.7 |
| Acetyl-POMC [205 – 220] | 1786.88 | 0.00768 | 9.12 | 19.57 | 6.62 | 5.46 | 0.5 |
| POMC [142 – 160] | 2229.92 | 0.0324 | 29.1 | 17.65 | 8.85 | 8.59 | 1.6 |
| | 887.9 | 0.0377 | 25.95 | 13.44 | 11.65 | 5.84 | 1.9 |
Table 4S. List of not-normally distributed peaks that are detected at significantly different levels in dehydrated (B)
aged and (D) adult rats.
| Peptide match | M+H, exp | p-value, Wilcoxon | Intensity B | Intensity D | StD-B | StD-D | Ratio B/D |
| --- | --- | --- | --- | --- | --- | --- | --- |
| Des-ac-a-MSH | 1622.78 | 0.00266 | 257.14 | 95.16 | 170.63 | 30.88 | 2.7 |
| POMC [126 - 136] | 1371.83 | 0.0038 | 156.49 | 48.78 | 90.89 | 27.15 | 3.2 |
| | 1938.71 | 0.00716 | 9.79 | 24.29 | 7.09 | 14.77 | 0.4 |
| Vasopressin-neurophysin 2-copeptin [155-168] | 1535.81 | 0.00908 | 28.95 | 12.57 | 13.61 | 5.37 | 2.3 |
| POMC [142-158] | 2019.84 | 0.0102 | 106.03 | 34.61 | 62.68 | 25.14 | 3.1 |
| | 2623.72 | 0.0102 | 2.81 | 4.3 | 2.26 | 1.59 | 0.7 |
| | 2543.73 | 0.0131 | 3.56 | 5.73 | 3.6 | 2.65 | 0.6 |
| | 3036.92 | 0.0131 | 2.23 | 12.73 | 4.14 | 24.16 | 0.2 |
| | 2565.7 | 0.0245 | 0.97 | 1.53 | 0.42 | 0.64 | 0.6 |
| | 2937.76 | 0.0245 | 1.15 | 3.19 | 1.28 | 5.11 | 0.4 |
| POMC [142 - 153] | 1513.98 | 0.0263 | 152.89 | 67.88 | 77.91 | 56.71 | 2.3 |
| ProSAAS [42-57] | 1514.8 | 0.0263 | 123.55 | 54.55 | 63.51 | 44.3 | 2.3 |
| | 2254.81 | 0.0263 | 30.37 | 11.72 | 22.37 | 8.68 | 2.6 |
| Phosphoserine-POMC [142-158] | 2099.8 | 0.0292 | 21.35 | 8.8 | 16.69 | 9.58 | 2.4 |
| | 1900.8 | 0.0479 | 25.49 | 44.21 | 35.06 | 33.72 | 0.6 |
| POMC [205 - 221] | 1858.82 | 0.0526 | 51.15 | 24.42 | 33.2 | 10.74 | 2.1 |

## Slide 3
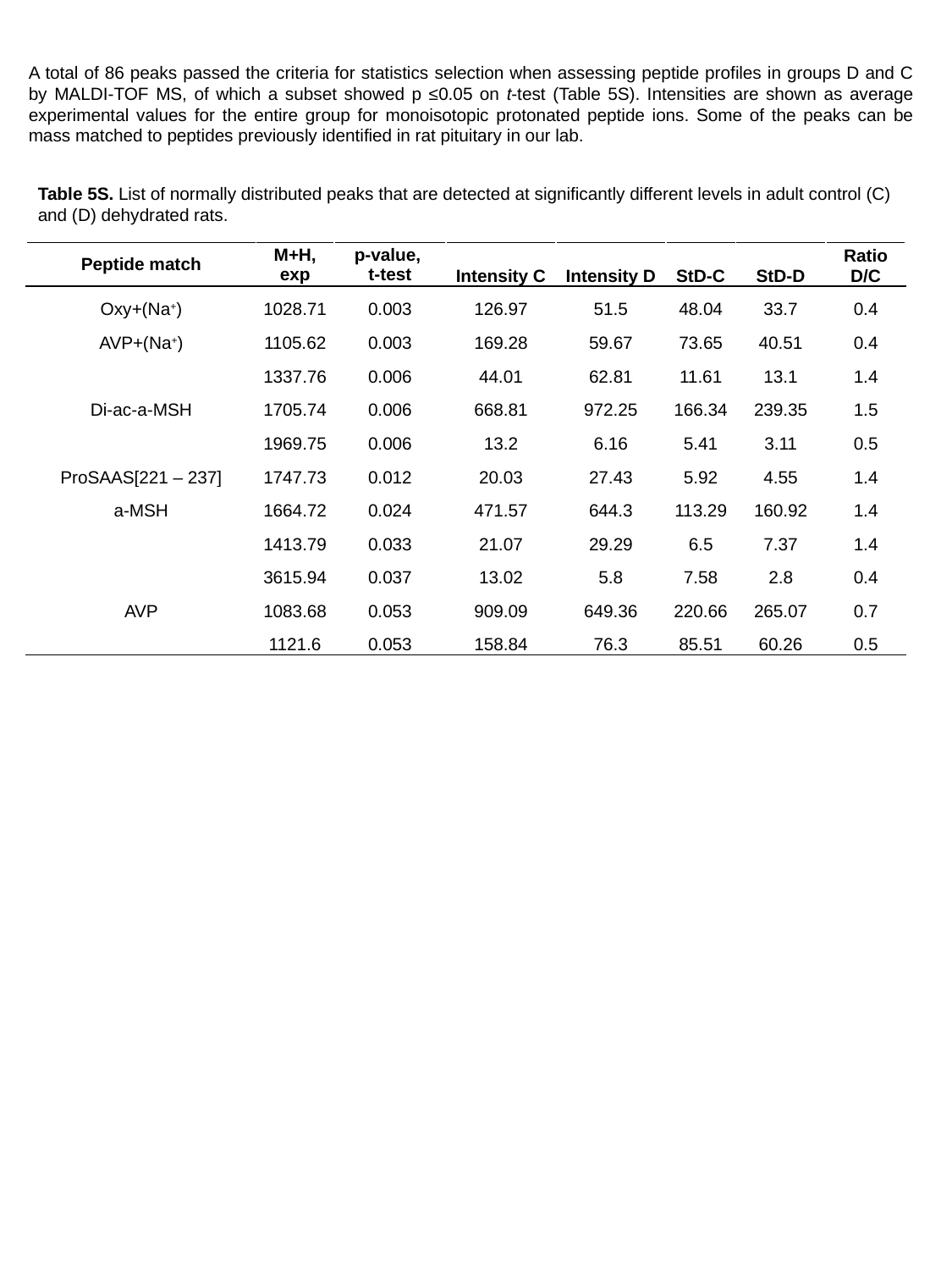

A total of 86 peaks passed the criteria for statistics selection when assessing peptide profiles in groups D and C by MALDI-TOF MS, of which a subset showed p ≤0.05 on t-test (Table 5S). Intensities are shown as average experimental values for the entire group for monoisotopic protonated peptide ions. Some of the peaks can be mass matched to peptides previously identified in rat pituitary in our lab.
Table 5S. List of normally distributed peaks that are detected at significantly different levels in adult control (C) and (D) dehydrated rats.
| Peptide match | M+H, exp | p-value, t-test | Intensity C | Intensity D | StD-C | StD-D | Ratio D/C |
| --- | --- | --- | --- | --- | --- | --- | --- |
| Oxy+(Na+) | 1028.71 | 0.003 | 126.97 | 51.5 | 48.04 | 33.7 | 0.4 |
| AVP+(Na+) | 1105.62 | 0.003 | 169.28 | 59.67 | 73.65 | 40.51 | 0.4 |
| | 1337.76 | 0.006 | 44.01 | 62.81 | 11.61 | 13.1 | 1.4 |
| Di-ac-a-MSH | 1705.74 | 0.006 | 668.81 | 972.25 | 166.34 | 239.35 | 1.5 |
| | 1969.75 | 0.006 | 13.2 | 6.16 | 5.41 | 3.11 | 0.5 |
| ProSAAS[221 – 237] | 1747.73 | 0.012 | 20.03 | 27.43 | 5.92 | 4.55 | 1.4 |
| a-MSH | 1664.72 | 0.024 | 471.57 | 644.3 | 113.29 | 160.92 | 1.4 |
| | 1413.79 | 0.033 | 21.07 | 29.29 | 6.5 | 7.37 | 1.4 |
| | 3615.94 | 0.037 | 13.02 | 5.8 | 7.58 | 2.8 | 0.4 |
| AVP | 1083.68 | 0.053 | 909.09 | 649.36 | 220.66 | 265.07 | 0.7 |
| | 1121.6 | 0.053 | 158.84 | 76.3 | 85.51 | 60.26 | 0.5 |
